# Supplementary material for: A multi-factor trafficking site on the spliceosome remodeling enzyme BRR2 recruits C9ORF78 to regulate alternative splicing
Source: Nat Commun. 2022 Mar 3;13:1132. doi: 10.1038/s41467-022-28754-2 (PMC8894380; doi:10.1038/s41467-022-28754-2)
Supplement: Supplementary file 1 — Supplementary Information [file 41467_2022_28754_MOESM1_ESM.pdf]

Supplementary Information

A multi-factor trafficking site on spliceosome remodeling enzyme, BRR2, recruits C9ORF78 to regulate alternative splicing

Alexandra Bergfort[1,§], Marco Preußner[2], Benno Kuropka[3,4], İbrahim Avşar Ilik[5], Tarek Hilal[1,4,6], Gert Weber[7], Christian Freund[3], Tuğçe Aktaş[5], Florian Heyd[2], Markus C. Wahl[1,7,*]

[1] Freie Universität Berlin, Institute of Chemistry and Biochemistry, Laboratory of Structural Biochemistry, Takustrasse 6, D-14195 Berlin, Germany

[2] Freie Universität Berlin, Institute of Chemistry and Biochemistry, Laboratory of RNA Biochemistry, Takustrasse 6, D-14195 Berlin, Germany

[3] Freie Universität Berlin, Institute of Chemistry and Biochemistry, Laboratory of Protein Biochemistry, Thielallee 63, D-14195, Berlin, Germany

[4] Freie Universität Berlin, Institute of Chemistry and Biochemistry, Core Facility BioSupraMol, Thielallee 63, D-14195, Berlin, Germany

[5] Max Planck Institute for Molecular Genetics, Ihnestr. 63, D-14195 Berlin, Germany

[6] Freie Universität Berlin, Institute of Chemistry and Biochemistry, Research Center of Electron Microscopy and Core Facility BioSupraMol, Fabeckstr. 36a, 14195 Berlin, Germany

[7] Helmholtz-Zentrum Berlin für Materialien und Energie, Macromolecular Crystallography, Albert-Einstein-Straße 15, D-12489 Berlin, Germany

§ Present address: Yale University, Molecular Biophysics and Biochemistry, 333 Cedar Street, New Haven, CT 06510, USA

* Correspondence to: markus.wahl@fu-berlin.de

Supplementary Tables

Supplementary Table 1: CryoEM data collection and refinement statistics

Complex	BRR2^{HR}-PRPF8^{Jab1}-C9ORF78	BRR2^{HR}-FBP21$^{200-376}$
**Data collection**		
Microscope	FEI Titan Krios G3i	
Voltage [keV]	300	
Camera	Falcon 3EC	
Magnification	120,000	96,000
Pixel size [Å/px]	0.657	0.832
Total electron exposure [e^{-}/Å^2]	40	40
Exposure rate [e^{-}/px/s]	0.6	0.7
No. of frames collected during exposure	33	33
Defocus range [µm]	0.8 -1.8	0.8 -1.8
Automation software	EPU2.10	EPU2.09
Micrographs collected	5,160	1,986
Micrographs used	4,986	1,877
**Data processing**		
Total extracted particles	936,716	787,610
Refined particles	542,565	271,493
Final particles	370,493	57,854
Point-group or helical symmetry parameters	C1	C1
Global resolution [Å]	2.72	3.30
$FSC_{0.143}$ unmasked/masked [Å]	3.1 / 2.7	3.7 / 3.0
Local resolution range [Å]	30.0 - 2.2	24.6 - 2.4
Map sharpening B factor [Å^2] / (B factor range)	-97	-120.3
Map sharpening method	Local B-factor	Local B-factor
**Model refinement**		
CC mask	0.89	0.86
CC volume	0.89	0.85
Model		
Non-H atoms	16,485	14,054
Protein residues	2,046	1,746
RMSD[1]		
Bond lengths [Å]	0.004	0.005
Bond angles [°]	0.564	0.602
Ramachandran plot		
Favored [%]	97.84	97.18
Allowed [%]	2.16	2.82
Outliers [%]	0	0
Model quality[2]		
Clash score	7.93	10.12
Rotamer outliers [%]	0.93	0.96
Overall score	1.47	1.68
Data availability		
EMDB ID	13046	13045
PDB ID	7OS2	7OS1

[1] RMSD, root-mean-square deviation from ideal geometry
[2] Assessed using MolProbity[67]

Supplementary Table 2: PCR primers

**C9ORF78 (RT-qPCR)**	
Forward	5'-TCCTTCGTGCCTACCAACAT-3'
Reverse	5'-GCTTCTCCGTGTCACCTACT-3'
**GAPDH (RT-qPCR)**	
Forward	5'-CTTCGCTCTCTGCTCCTCCTGTTCG-3'
Reverse	5'-ACCAGGCGCCCAATACGACCAAAT-3'
**PTBP2 (radioactive RT-PCR)**	
Forward	5'-CTGAGTCCTTTGGCCATTCC-3'
Reverse	5'-CACGCTGCACATCTCCATAA-3'
**SMARCA4 (radioactive RT-PCR)**	
Forward	5'-AAACTCTCCCCTAACCCACC-3'
Reverse	5'-TGGATGAAGACCTCGCTGAG-3'
**C1ORF131 (radioactive RT-PCR)**	
Forward	5'-ACAAGTAGAAGTGGTAGAATTTCAC-3'
Reverse	5'-TCCACATCTCTCTCCAAAACAC-3'

Supplementary Figures

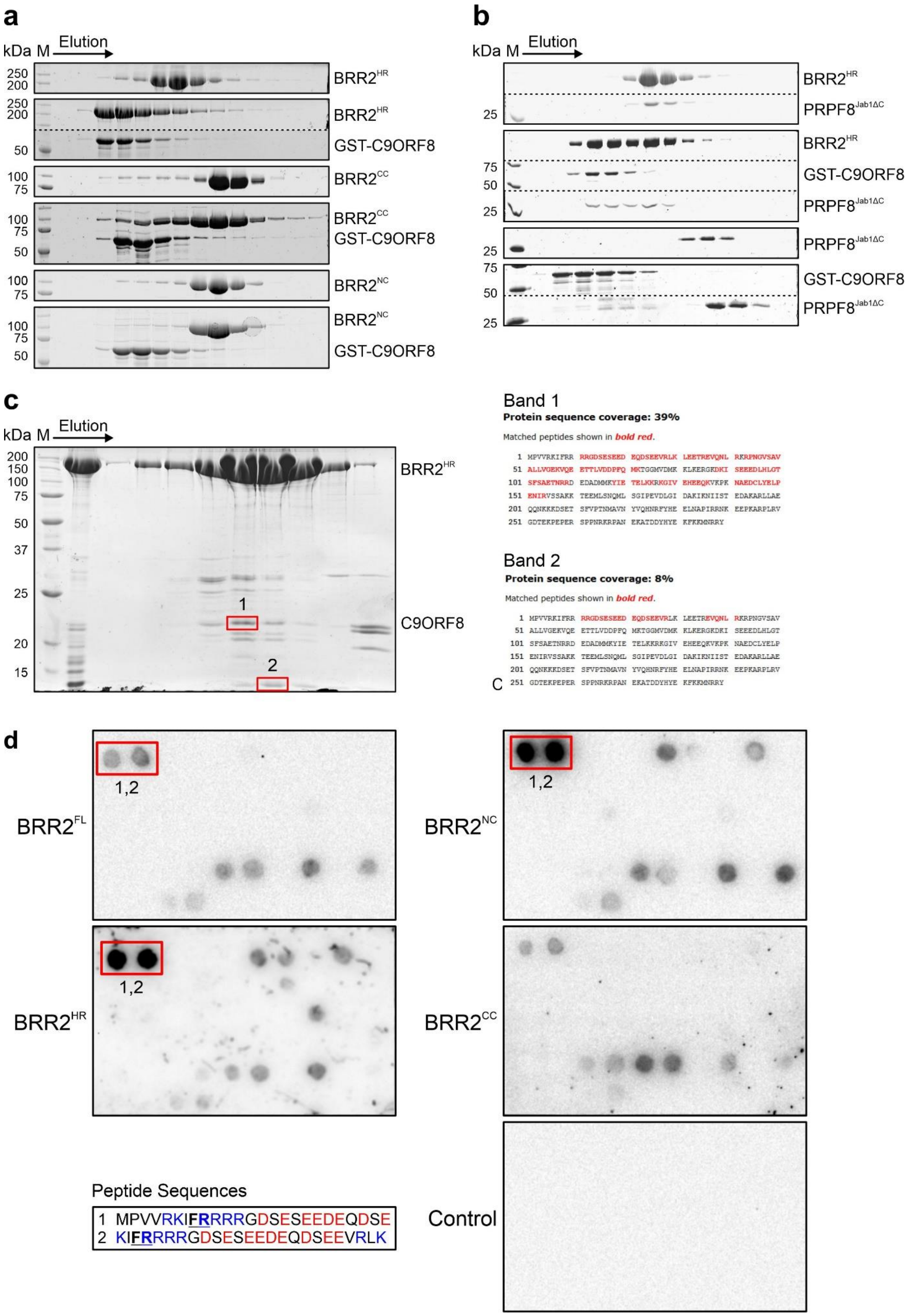

Supplementary Figure 1: Protein interaction studies in vitro

a, SDS-PAGE gels showing elution fractions from analytical SEC, monitoring interaction of GST-C9ORF78 with BRR2 truncation variants (BRR2^{HR}, BRR2^{NC}, BRR2^{CC}). Elution direction is indicated by an arrow. The same elution fractions from runs under identical conditions are shown. Protein bands are identified on the right. M, molecular mass marker. In the second panel, upper and lower regions of the same gels were spliced together. Dotted line, splice position. Independent experiments were conducted twice with similar results.

b, Like **a**, monitoring simultaneous interaction of PRPF8$^{Jab1\Delta C}$ and C9ORF78 with BRR2^{HR} (upper panels), and interaction of C9ORF78 with PRPF8$^{Jab1\Delta C}$ in the absence of BRR2^{HR} (lower panels). Elution direction is indicated by an arrow. The same elution fractions from runs under identical conditions are shown. Protein bands are identified on the right. M, molecular mass marker. In the first, second and fourth panel, different regions of the same gels were spliced together. Dotted lines, splice positions. Independent experiments were conducted twice with similar results. Source data for **a** and **b** are provided as a Source Data file.

c, Left, SDS-PAGE gels showing elution fractions from analytical SEC after digest of the BRR2^{HR}-C9ORF78 complex with elastase. Elution direction is indicated by an arrow. Protein bands are identified on the right. M, molecular mass marker. IS, injected sample. Gel bands subjected to mass spectrometric analysis are numbered and highlighted by red boxes. Right, C9ORF78 peptides and sequence coverage identified in the analyzed gel bands by mass spectrometry. Independent experiments were conducted twice with similar results.

d, C9ORF78 peptide SPOT arrays incubated with His-tagged BRR2 constructs and probed by anti-His antibody. Peptides 1 and 2 (red boxes) are highly reactive with BRR2^{FL}, BRR2^{HR} and BRR2^{CC}, but not with BRR2^{NC}. Sequences for peptides 1 and 2 are indicated in the lower left box. The conserved BRR2-interacting F8-R9 motif, contained in these peptides, is shown in bold and underlined. Positively charged residues, blue; negatively charged residues, red.

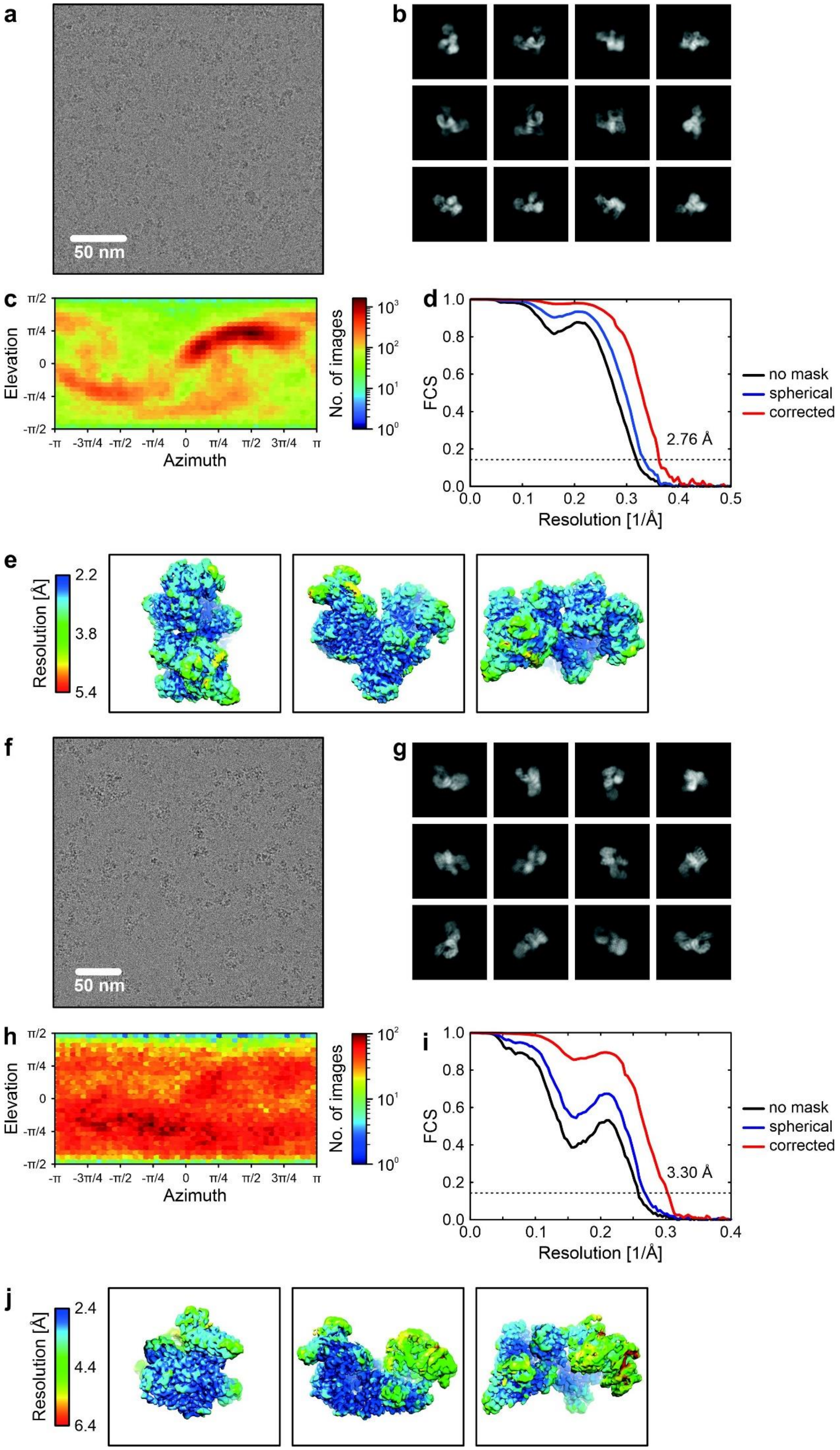
a
50 nm
b
c
Elevation
Azimuth
No. of images
d
FCS
Resolution [1/Å]
no mask
spherical
corrected
2.76 Å
e
Resolution [Å]
2.2
3.8
5.4
f
50 nm
g
h
Elevation
Azimuth
No. of images
i
FCS
Resolution [1/Å]
no mask
spherical
corrected
3.30 Å
j
Resolution [Å]
2.4
4.4
6.4

65

Supplementary Figure 2: CryoEM analysis of BRR2^{HR}-PRP8^{Jab1}-C9ORF78 and BRR2^{HR}-FBP21$^{200\text{-}376}$ complexes

a, Representative cryoEM micrographs of the BRR2^{HR}-PRP8^{Jab1}-C9ORF78 complex. Scale bar, 50 nm. 5,160 micrographs were recorded from the same sample, particle images were picked from 4,986 high-quality micrographs.

b, 2D class averages of BRR2^{HR}-PRP8^{Jab1}-C9ORF78 particle images after reference-free classification.

c, Viewing direction distribution plot of the particle images used for the final reconstruction of the BRR2^{HR}-PRP8^{Jab1}-C9ORF78 cryoEM map as obtained during NU refinement with cryoSPARC.

d, Global resolution estimation for the BRR2^{HR}-PRP8^{Jab1}-C9ORF78 cryoEM map by gold-standard Fourier shell correlation (FSC). Dashed line, FSC$_{0.143}$.

e, Local resolution estimation as determined with cryoSPARC, ranging from 2.2 Å to 5.4 Å for the BRR2^{HR}-PRP8^{Jab1}-C9ORF78 cryoEM map.

f, Representative cryoEM micrographs of the BRR2^{HR}-FBP21$^{200\text{-}376}$ complex. Scale bar, 50 nm. 1,986 micrographs were recorded from the same sample, particle images were picked from 1,877 high-quality micrographs.

g, 2D class averages of BRR2^{HR}-FBP21$^{200\text{-}376}$ particle images after reference-free classification.

h, Viewing direction distribution plot of the particle images used for the final reconstruction of the BRR2^{HR}-FBP21$^{200\text{-}376}$ cryoEM map as obtained during NU refinement with cryoSPARC.

i, Global resolution estimation for the BRR2^{HR}-FBP21$^{200\text{-}376}$ cryoEM map by gold-standard Fourier shell correlation (FSC). Dashed line, FSC$_{0.143}$.

j, Local resolution estimation as determined with cryoSPARC, ranging from 2.4 Å to 6.4 Å for the BRR2^{HR}-FBP21$^{200\text{-}376}$ cryoEM map.

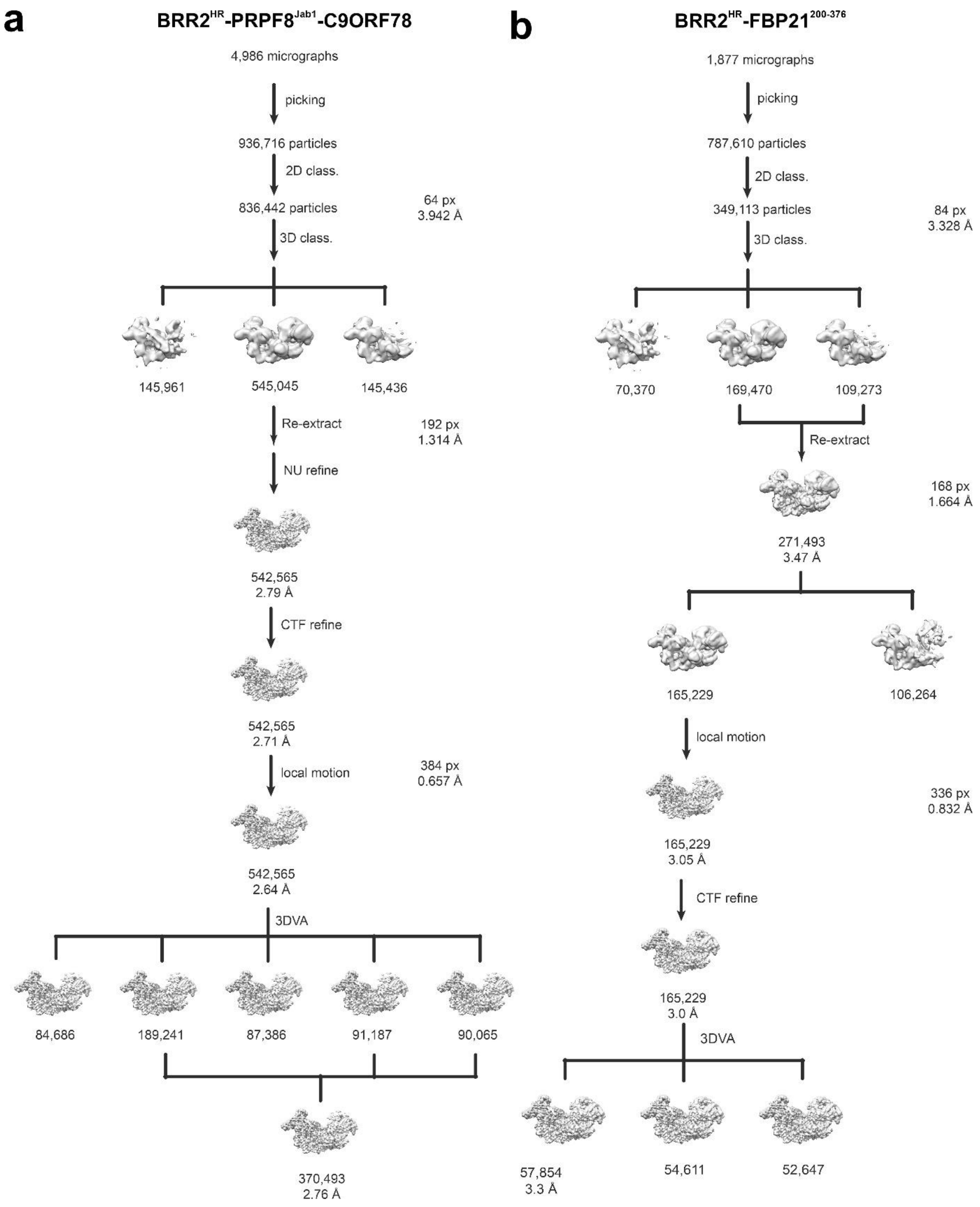

Supplementary Figure 3: CryoEM data refinement.

a, Reconstruction of the BRR2^{HR}-PRPF8^{Jab1}-C9ORF78 cryoEM map. 936,716 particle images were picked from 4,986 micrographs and subjected to reference-free 2D classification. 836,442 particle images were selected for heterogeneous 3D refinement into 3 classes. The best appearing class, consisting of 545,045 particle images, was selected for re-extraction with

a box size of 384 px, Fourier-cropped to 192 px (pixel size 1.314 Å). Homogeneous NU refinement yielded a reconstruction at 2.79 Å resolution that could be improved to 2.71 Å resolution by CTF refinement. To further improve the reconstruction, local motion correction was applied and particle images were re-extracted at full spatial resolution with a box size of 384 px (0.657 Å/px). NU refinement yielded a reconstruction at 2.64 Å resolution that showed only fragmented density for C9ORF78. 3D variability analysis using a generous mask covering the N-terminal part of C9ORF78 was applied to remove sub-stoichiometric complexes. 370,493 particle images were selected for final NU refinement yielding a reconstruction at 2.76 Å resolution.

b, Reconstruction of the BRR2^{HR}-FBP21$^{200\text{-}376}$ cryoEM map. 787,610 particle images were picked from 1,877 micrographs and subjected to reference-free 2D classification. 349,113 particle images were selected for heterogeneous 3D refinement into 3 classes. Two classes were merged, the 271,494 particle images were re-extracted with a box size of 336 px, Fourier-cropped to 168 px (pixel size 1.664 Å). Homogeneous refinement yielded a reconstruction of 3.47 Å that was subjected to another round of heterogeneous 3D refinement. 165,229 particle images were selected for local motion correction and un-binned re-extraction (box size 336 px, 0.832 Å/px), which yielded a map at a resolution of 3.0 Å. 3D variability analysis using a mask around the FBP21$^{200\text{-}376}$-binding region was applied to select the final 57,854 particle images for NU refinement, yielding a reconstruction at 3.30 Å resolution.

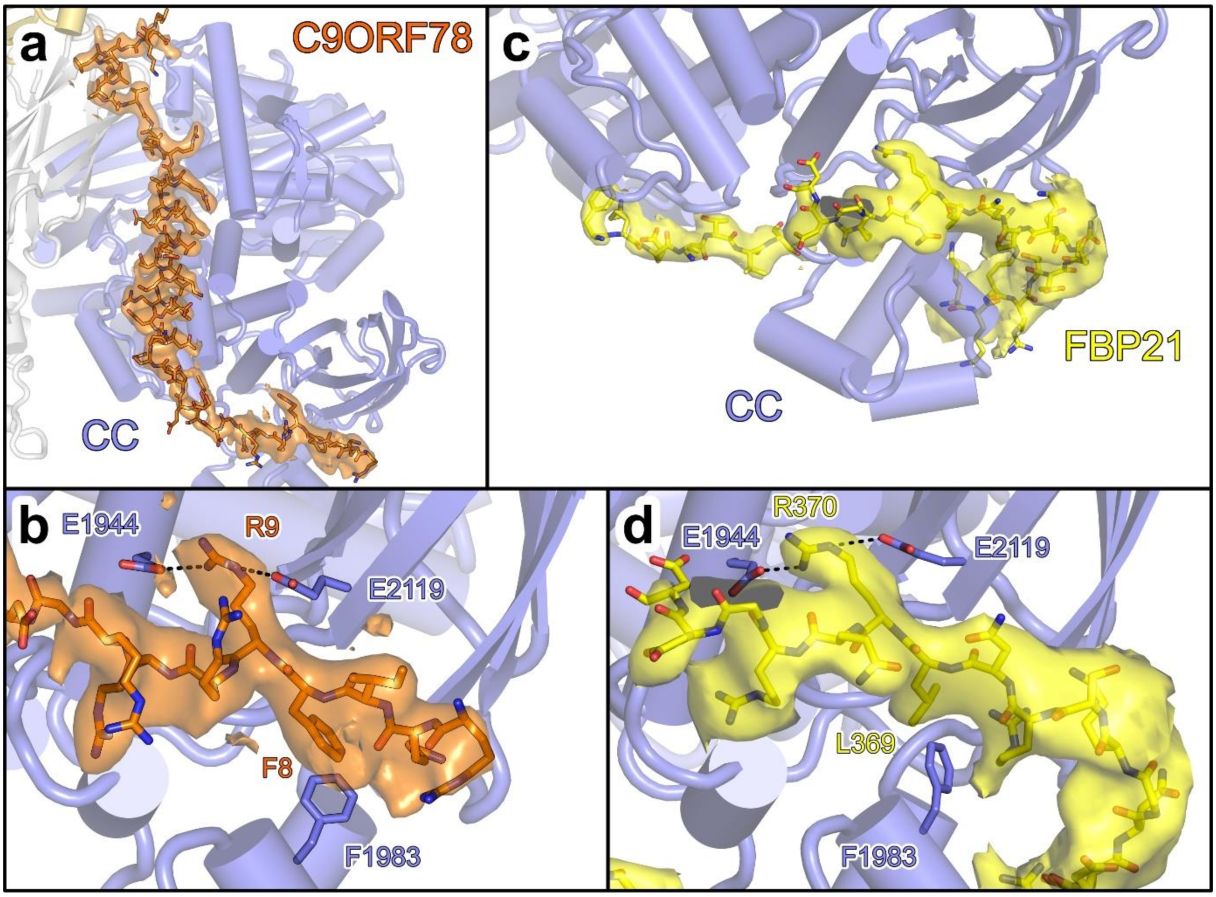

Supplementary Figure 4: Details of cryoEM maps.

a-**d**, CryoEM maps as shown in Fig. 1c,d of a BRR2^{HR}-PRPF8^{Jab1}-C9ORF78 complex (**a**, **b**) and of a BRR2^{HR}-FBP21$^{200\text{-}376}$ complex (**c**, **d**), contoured at > 10 RMSD and covering the C9ORF78 or FBP21$^{200\text{-}376}$ molecules, respectively. BRR2 is shown as a cartoon, C9ORF78 or FBP21$^{200\text{-}376}$ are shown as sticks. The close-up views (**b**, **d**) zoom in on the common BRR2-interacting motifs (C9ORF78, F8-R9; FBP21$^{200\text{-}376}$, L369-R370).

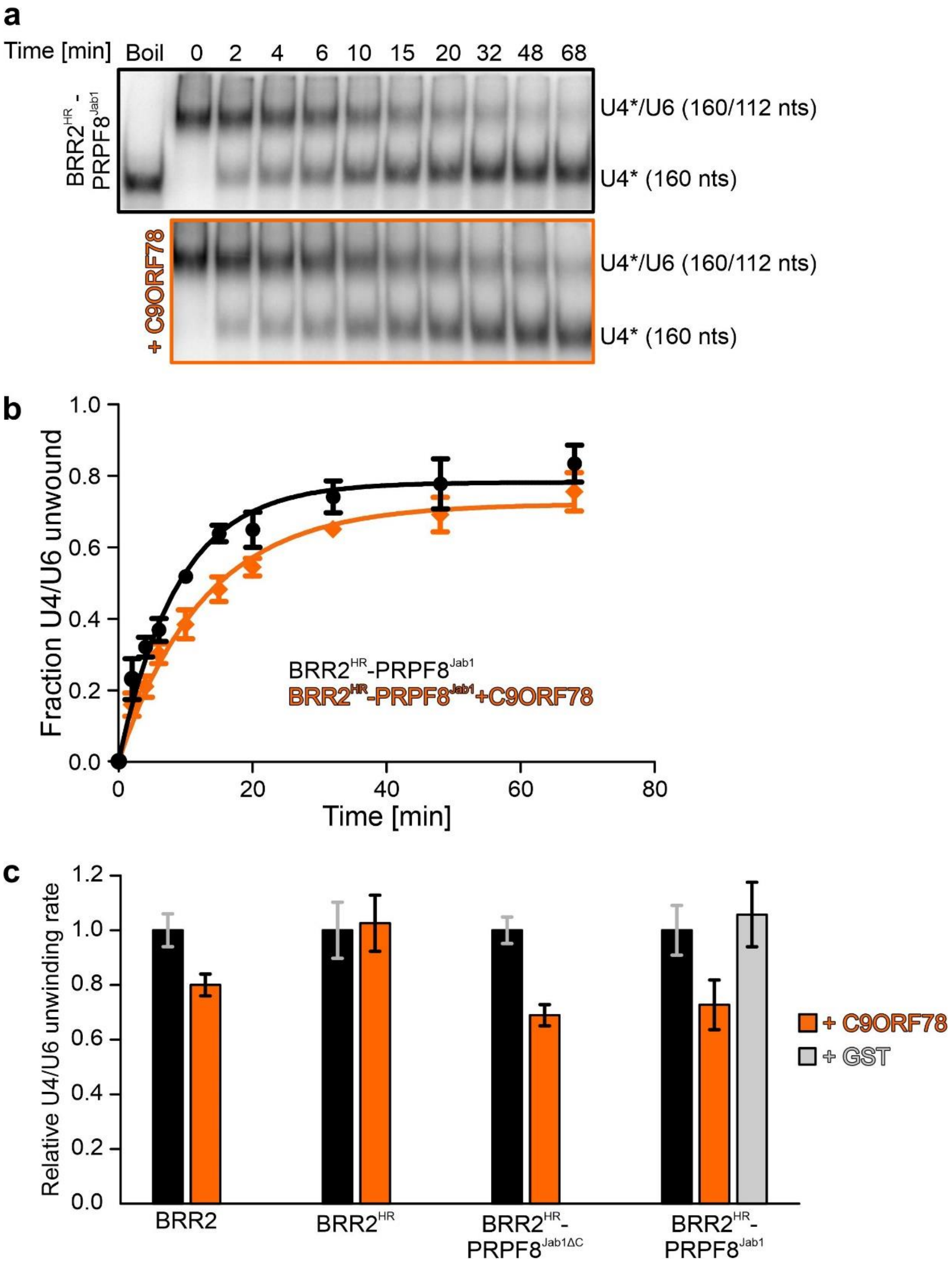

Supplementary Figure 5: C9ORF78 has a moderate inhibitory effect on the BRR2 helicase activity.

a, Exemplary non-denaturing PAGE, monitoring BRR2^{HR}-PRPF8^{Jab1}-mediated U4/U6 unwinding in the absence (black outline) or presence (orange outline) C9ORF78. Bands are identified on the right with numbers of nucleotides (nts). U4*, radio-labeled U4 snRNA.

b, Quantification of data shown in **a**. Data points represent means ± SD; n = 3 technical replicates. Data were fit to a single exponential equation (fraction unwound = A (1 – exp[- k_ut])); A, amplitude of the reaction; k_u, apparent first-order rate constant of unwinding; t, time).

c, Change of unwinding rate of the indicated BRR2 variants alone or in complex with $PRPF8^{Jab1\Delta C}$ or $PRPF8^{Jab1}$ in the absence (black bars) or presence (orange bars) C9ORF78. Gray bar, control with GST instead of C9ORF78 added. Bars indicate means ± SD obtained from curve fits to all n = 3 technical replicates, each for ten time points. Source data for **b** and **c** are provided as a Source Data file.

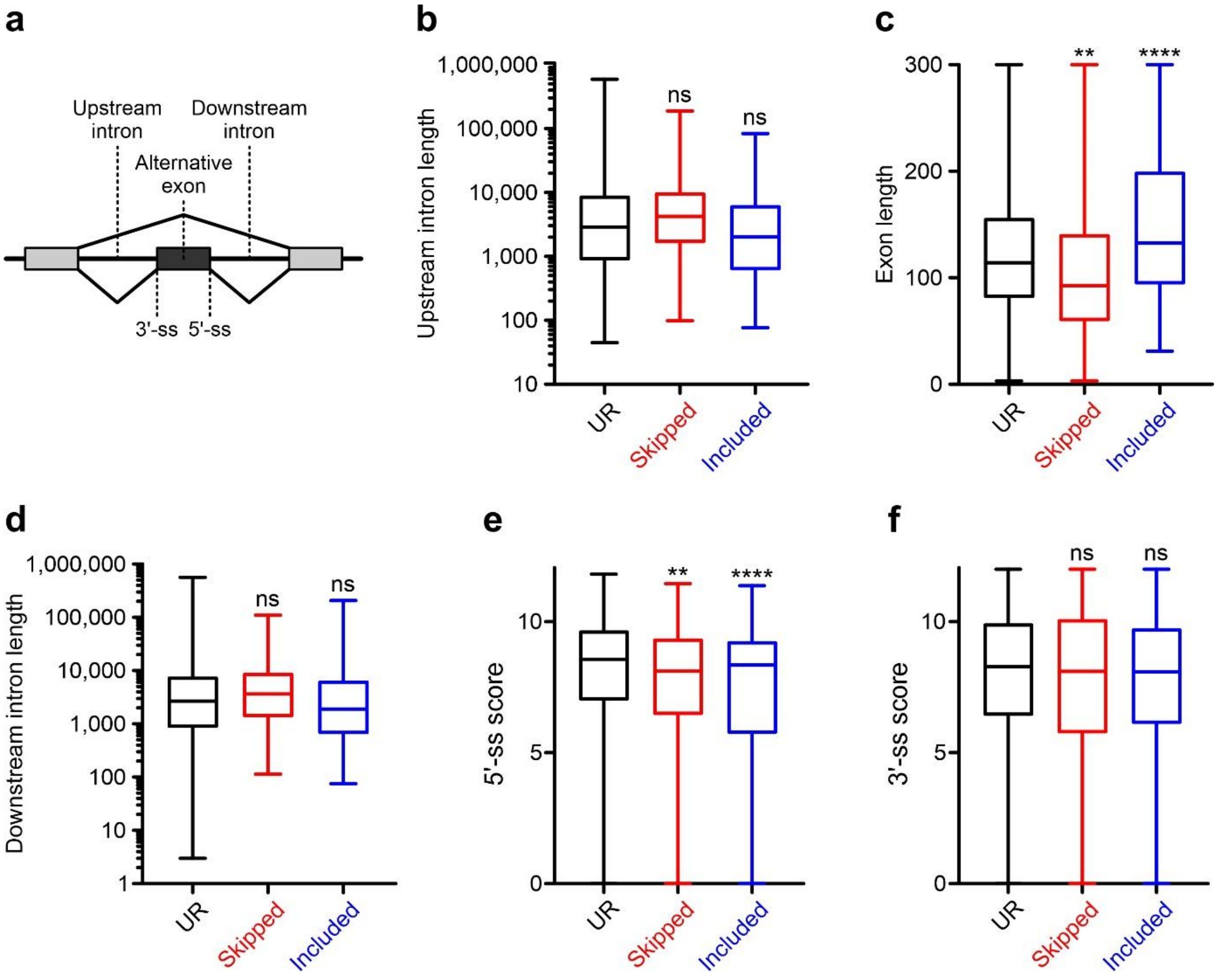

Supplementary Figure 6: Features of alternative exons regulated by C9ORF78.

a, Scheme for alternative exons.

b-f, Features of skipped exons regulated by C9ORF78. In each panel, unregulated (UR) exons quantified by rMATS (black; n = 122,362) are compared to alternative exons showing C9ORF78 KD-induced skipping (red; n = 196) or C9ORF78 KD-induced inclusion (blue; n = 194). Horizontal lines, medians; whiskers, minimum and maximum values; boxes, upper and lower quartile. Statistical significance was determined by unpaired, two-sided Student's t-tests (**, $p < 0.01$; ****, $p < 0.0001$; ns, not significant).

b, Lengths of the upstream introns.

c, Lengths of the alternative exons; **, $p = 0.0027$; ****, $p < 0.0001$.

d, Lengths of the downstream introns.

e, Strengths of the 5'-splice sites of the alternative exons (5'-ss); **, $p = 0.0054$; ****, $p < 0.0001$.

f, Strengths of the 3'-splice sites of the alternative exons (3'-ss).

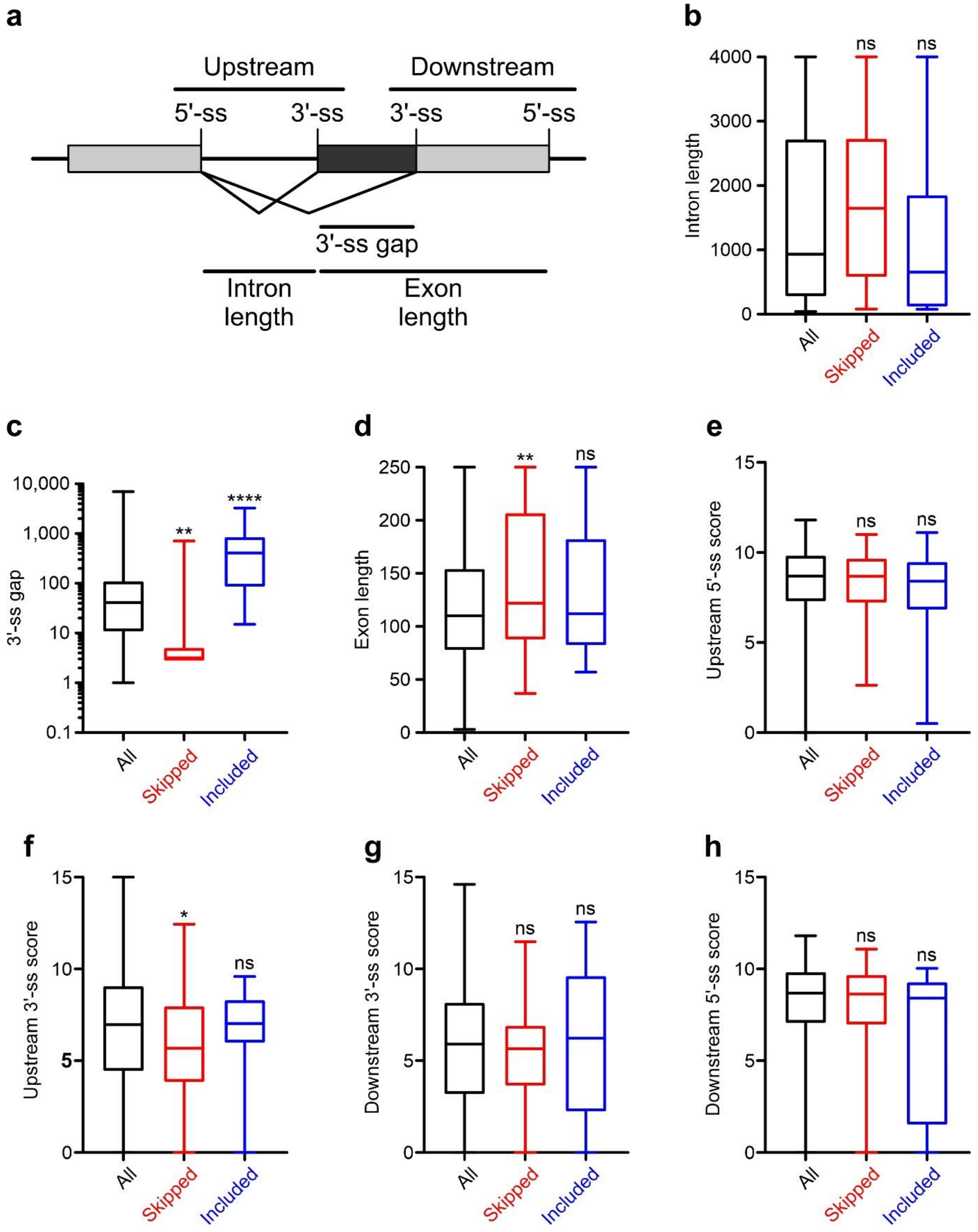

Supplementary Figure 7: Features of alternative 3'-ss regulated by C9ORF78.

a, Scheme for alternative 3'-ss usage.

b-h, Features of alternative 3'-ss regulated by C9ORF78. In each panel, all alternative 3'-ss quantified by rMATS (black; n = 6,160) are compared to alternative 3'-ss showing C9ORF78

KD-induced skipping (red; $n = 89$) and alternative 3'-ss showing C9ORF78 KD-induced inclusion (blue; $n = 16$). Horizontal lines, medians; whiskers, minimum and maximum values; boxes, upper and lower quartile. Statistical significance was determined by unpaired, two-sided Student's t-tests (*, $p < 0.05$; **, $p < 0.01$; ****, $p < 0.0001$; ns, not significant).

b, Lengths of the upstream introns.

c, Distances between the upstream 3'-ss and the downstream 3'-ss (3'-ss gap); **, $p = 0.0084$; ****, $p < 0.0001$.

d, Lengths of the exons containing the alternative 3'-ss; **, $p = 0.0095$.

e, Strengths of the 5'-splice sites of the upstream introns (upstream 5'-ss).

f, Strengths of the upstream 3'-splice sites; *, $p = 0.0176$.

g, Strengths of the downstream 3'-splice sites.

h, Strengths of the 5'-splice sites of the downstream introns (downstream 5'ss).

Upstream 3'-ss requiring C9ORF78 for usage are characterized by a very short distance to the alternative downstream 3'-ss (**c**), a comparatively long exon that contains the alternative 3'-ss (**d**), and they are comparatively weak (**f**).

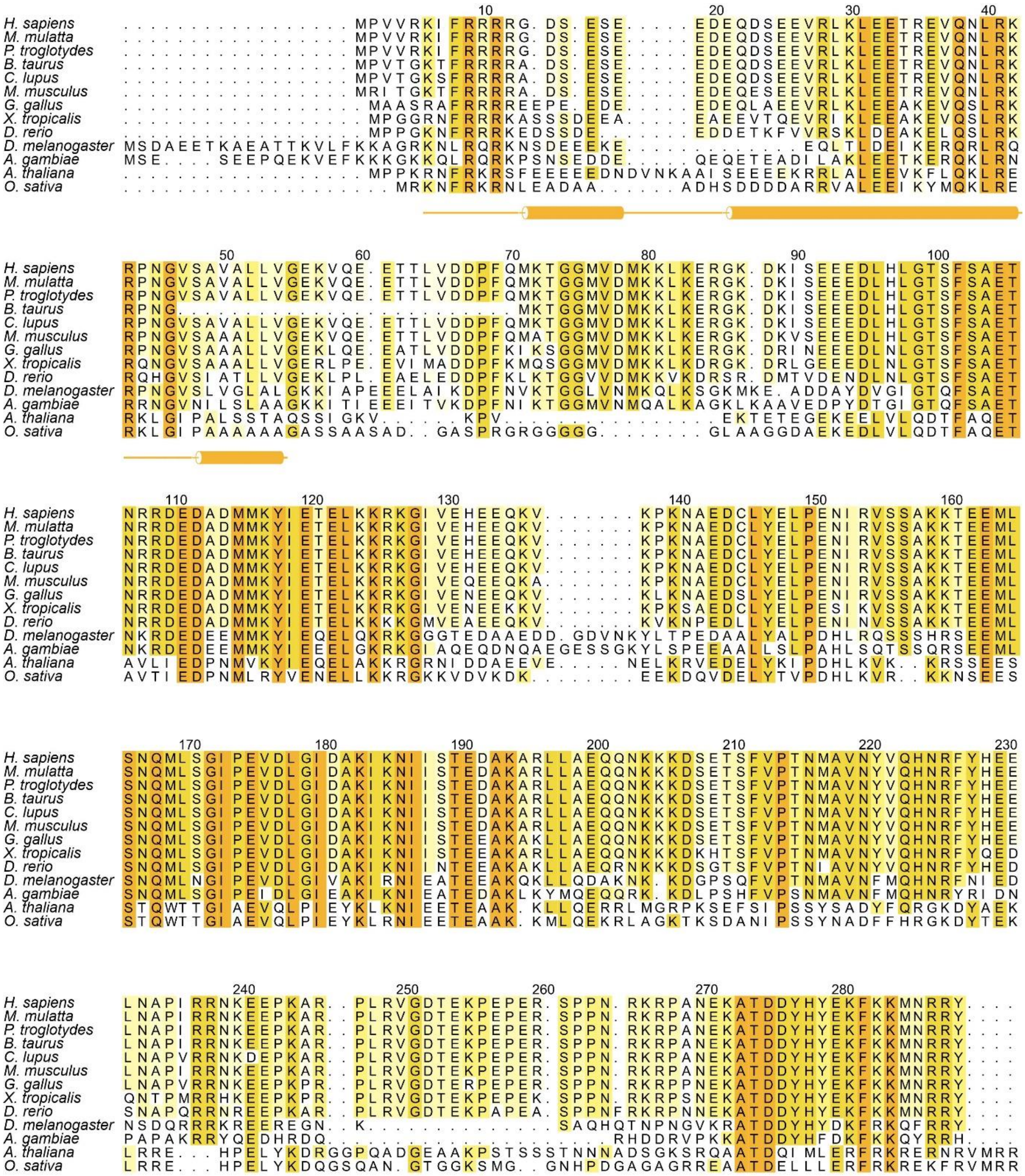

Supplementary Figure 8: C9ORF78 conservation.

Multiple sequence alignment of C9ORF78 from different species, as indicated on the left. Residues are colored light to dark according to increasing level of conservation (54 % conservation, light yellow; 100 % conservation, orange). Residue numbering according to human C9ORF78 is indicated above the sequences. Secondary structure elements observed in the BRR2^{HR}-PRPF8^{Jab1}-C9ORF78 cryoEM structure are indicated below the sequences. The

alignment was prepared with Homologene (NCBI) employing Clustal Omega[75] and shaded with ALSCRIPT[76].

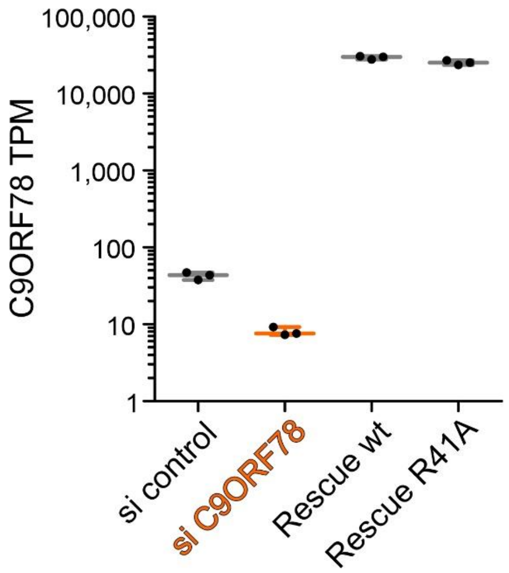

Supplementary Figure 9: Knock-down and over-expression.

RNA-seq data confirming KD of endogenous C9ORF78 and comparable levels of over-expression of siRNA-resistant C9ORF78^{wt} and C9ORF78^{R41A}. Horizontal lines, medians; whiskers, minimum and maximum values (partially hidden due to the low variations); n = 3 technical replicates. TPM, transcripts per million.

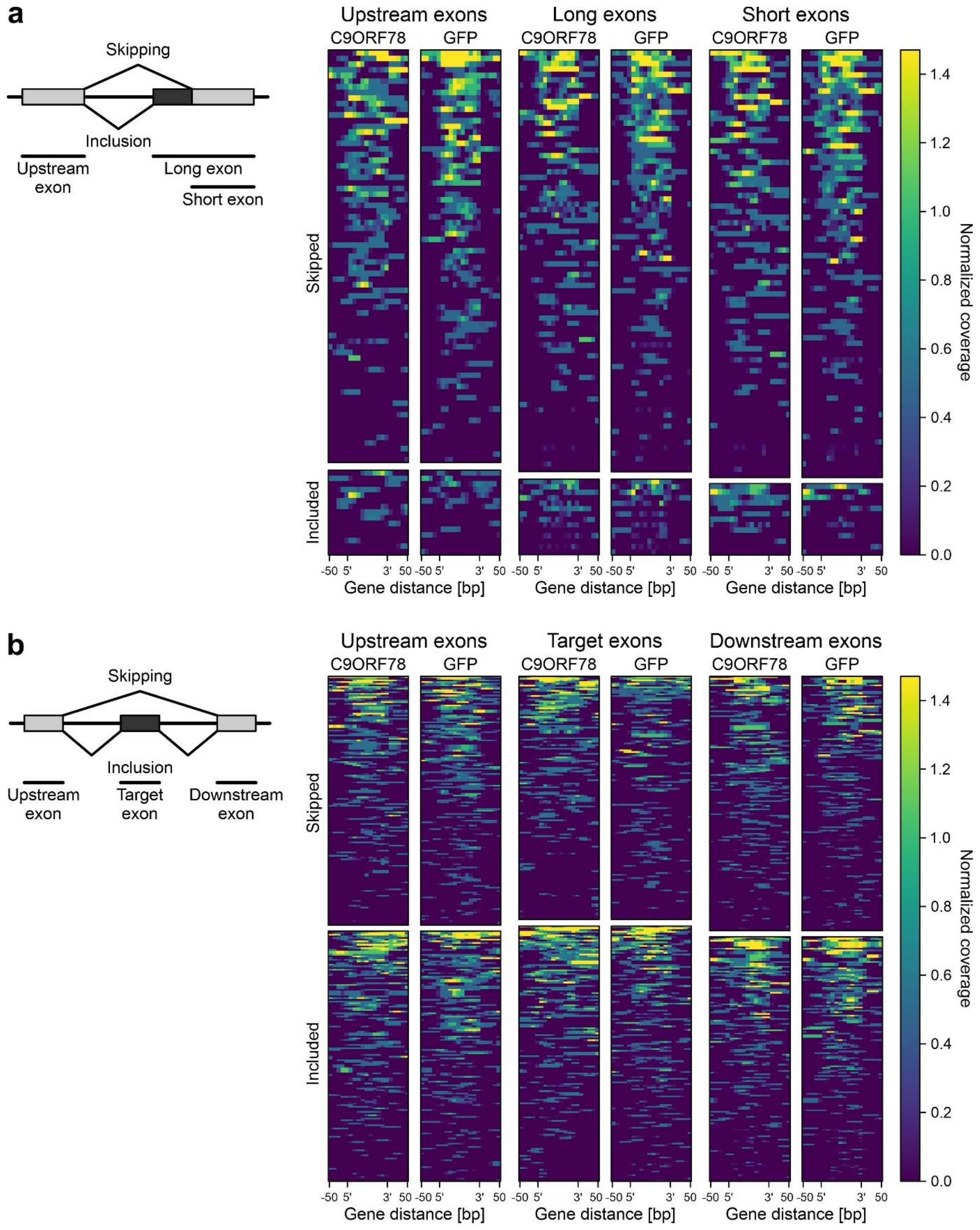

Supplementary Figure 10: Comparison of siRNA KD and FLASH data.

a, Cumulative coverage plots obtained by deepTools, displaying RNAs that show changes in alternative 3'-ss usage upon C9ORF78 KD and UV-crosslinks to C9ORF78 or GFP (negative control; FLASH-derived sequence coverage of C9ORF78 splicing targets detected by rMATS

analysis). Heat maps are colored by normalized coverage average (n = 2 biologically independent experiments). Sequences of upstream exons as well as long and short exons harboring the alternative 3'-ss (scheme on the left) plus 50 base pairs upstream and downstream of the exons (illustrated below the heat maps) were analyzed. Top, targets with upstream 3'-ss more skipped upon C9ORF78 KD; bottom, targets with upstream 3'-ss more included upon C9ORF78 KD.

b, Like **a**, but for skipped exon events.
