# Supplementary material for: A multi-factor trafficking site on the spliceosome remodeling enzyme BRR2 recruits C9ORF78 to regulate alternative splicing
Source: Nat Commun. 2022 Mar 3;13:1132. doi: 10.1038/s41467-022-28754-2 (PMC8894380; doi:10.1038/s41467-022-28754-2)
Supplement: Supplementary file 3 — Description of Additional Supplementary Files [file 41467_2022_28754_MOESM3_ESM.pdf]

Description of Additional Supplementary Files

File Name: Supplementary Data 1
Description: **RNA-seq data.** RNA-seq results upon C9ORF78 KD

File Name: Supplementary Data 2
Description: **MS-based proteomics results.** Proteins enriched in Flag-C9ORF78^{wt} IP or Flag-C9ORF78^{R41A} IP
