# Supplementary material for: A multi-factor trafficking site on the spliceosome remodeling enzyme BRR2 recruits C9ORF78 to regulate alternative splicing
Source: Nat Commun. 2022 Mar 3;13:1132. doi: 10.1038/s41467-022-28754-2 (PMC8894380; doi:10.1038/s41467-022-28754-2)
Supplement: Supplementary file 6 — Reporting Summary [file 41467_2022_28754_MOESM6_ESM.pdf]

nature research

Corresponding author(s): Markus C. Wahl

Last updated by author(s): Feb 4, 2022

Reporting Summary

Nature Research wishes to improve the reproducibility of the work that we publish. This form provides structure for consistency and transparency in reporting. For further information on Nature Research policies, see our Editorial Policies and the Editorial Policy Checklist.

Statistics

For all statistical analyses, confirm that the following items are present in the figure legend, table legend, main text, or Methods section.

n/a	Confirmed	
[ ]	[x]	The exact sample size (*n*) for each experimental group/condition, given as a discrete number and unit of measurement
[ ]	[x]	A statement on whether measurements were taken from distinct samples or whether the same sample was measured repeatedly
[ ]	[x]	The statistical test(s) used AND whether they are one- or two-sided<br>*Only common tests should be described solely by name; describe more complex techniques in the Methods section.*
[x]	[ ]	A description of all covariates tested
[x]	[ ]	A description of any assumptions or corrections, such as tests of normality and adjustment for multiple comparisons
[ ]	[x]	A full description of the statistical parameters including central tendency (e.g. means) or other basic estimates (e.g. regression coefficient) AND variation (e.g. standard deviation) or associated estimates of uncertainty (e.g. confidence intervals)
[ ]	[x]	For null hypothesis testing, the test statistic (e.g. *F*, *t*, *r*) with confidence intervals, effect sizes, degrees of freedom and *P* value noted<br>*Give P values as exact values whenever suitable.*
[x]	[ ]	For Bayesian analysis, information on the choice of priors and Markov chain Monte Carlo settings
[x]	[ ]	For hierarchical and complex designs, identification of the appropriate level for tests and full reporting of outcomes
[x]	[ ]	Estimates of effect sizes (e.g. Cohen's *d*, Pearson's *r*), indicating how they were calculated

Our web collection on statistics for biologists contains articles on many of the points above.

Software and code

Policy information about availability of computer code

Data collection	EPU, version 2.14 (SPA acquisition; referenced in text)

Data analysis	BBmap, version 38.72 (genome mapping of FLASH data)<br>Bedtools version 2.27.1 (extraction of splice sequences)<br>Bowtie2, version 2.3.5 (genome mapping of FLASH data)<br>Coot, version 0.8.9.1 (model building; referenced in text)<br>cryoSPARC, version 2.12.4 (all image analysis; referenced in text)<br>deepTools2 package, version 3.3.1 (cumulative coverage plots; referenced in text)<br>Excel, Microsoft Office Professional Plus 2016 (statistics; referenced in in text)<br>GraphPad Prism, version 5 (referenced in text)<br>Image-Quant, version 5.2 (quantification of bands on gels; referenced in text)<br>MaxQuant software package, version 1.6.14 (MS analysis; referenced in text)<br>Molprobity, version 4.5.1 (structure evaluation; referenced in text)<br>Perseus, version 1.6.14 (statistical analysis of MS data; reference 56)<br>PHENIX, version 1.17.1 (real space refinement; referenced in text)<br>PyMOL, version 1.8 (rmsd calculation; structure figure preparation; referenced in text)<br>rMATS, version 3.1.0 (calculation of alternative splicing changes)<br>STAR, version 2.7.9a (RNAseq data alignment, KD and FLASH experiments; referenced in text)<br>TEtools (SnakePipes), version 2.5.0 (enrichment plots, referenced in text)<br>umi-tools, version 1.0.0 (removal of PCR duplicates in FLASH data)

For manuscripts utilizing custom algorithms or software that are central to the research but not yet described in published literature, software must be made available to editors and reviewers. We strongly encourage code deposition in a community repository (e.g. GitHub). See the Nature Research guidelines for submitting code & software for further information.

Data

Policy information about availability of data

All manuscripts must include a data availability statement. This statement should provide the following information, where applicable:

- Accession codes, unique identifiers, or web links for publicly available datasets
- A list of figures that have associated raw data
- A description of any restrictions on data availability

CryoEM maps have been deposited in the Electron Microscopy Data Bank (https://www.ebi.ac.uk/pdbe/emdb) under accession codes EMD-13046 (BRR2HR-PRPF8Jab1-C9ORF78; https://www.ebi.ac.uk/pdbe/entry/emdb/EMD-13046) and EMD-13045 (BRR2HR-FBP21200-376; https://www.ebi.ac.uk/pdbe/entry/emdb/EMD-13045). Structure coordinates have been deposited in the RCSB Protein Data Bank (https://www.rcsb.org) with accession codes 7OS2 (BRR2HR-PRPF8Jab1-C9ORF78; https://www.rcsb.org/structure/7OS2)72 and 7OS1 (BRR2HR-FBP21200-376; https://www.rcsb.org/structure/7OS1)73. RNA sequencing data from siRNA KD and rescue experiments have been deposited in Gene Expression Omnibus (GEO; https://www.ncbi.nlm.nih.gov/geo) under accession codes GSE176517 (https://www.ncbi.nlm.nih.gov/geo/query/acc.cgi?acc=GSE176517) and GSE189362 (https://www.ncbi.nlm.nih.gov/geo/query/acc.cgi?acc=GSE189362). FLASH data have been deposited in GEO under accession code GSE176464 (https://www.ncbi.nlm.nih.gov/geo/query/acc.cgi?acc=GSE176464). The mass spectrometry proteomics data have been deposited in the ProteomeXchange Consortium (http://www.proteomexchange.org) via the PRIDE74 partner repository (https://www.ebi.ac.uk/pride/) under dataset identifier PXD031482 (https://www.ebi.ac.uk/pride/archive/projects/PXD031482). The reference proteome for the analysis of the mass spectrometry proteomics data was obtained from Uniprot (https://www.uniprot.org/proteomes/UP000005640). All other data are contained in the manuscript or the Supplementary Information. Source data are provided with this paper.

Field-specific reporting

Please select the one below that is the best fit for your research. If you are not sure, read the appropriate sections before making your selection.

[x] Life sciences [ ] Behavioural & social sciences [ ] Ecological, evolutionary & environmental sciences

For a reference copy of the document with all sections, see nature.com/documents/nr-reporting-summary-flat.pdf

Life sciences study design

All studies must disclose on these points even when the disclosure is negative.

Sample size	Sample sizes are provided in the Methods. Sample sizes were determined empirically or based on similar data reported in other scientific publications.<br>For cryoEM analyses, sample sizes were designed to yield a large number of particle images on the grids while avoiding non-specific aggregation.<br>For biochemical and biophysical assays, sample sizes were designed to provide significant and reproducible signals (analytical SEC; helicase activity assay, peptide SPOT, limited proteolysis) or to provide clearly visible and quantifiable bands on gels (SDS PAGE analyses of analytical SEC, helicase activity assays, radioactive RT-PCRs).
Data exclusions	Structural analysis by cryoEM involved the sorting of high quality particle images and rejection of poor quality particle images. Poor quality particle images may be due to compositional heterogeneity in the sample or may originate from particles being damaged in the process of grid preparation, e.g. at the air-water interface. The processing steps involving data exclusion are outlined in Supplementary Figure 3.<br>For other experiments, no data were excluded from the analyses.
Replication	The numbers of technical and biological replicates are indicated in the text and figure legends.<br>For gel analyses of helicase assays with subsequent quantification of data, at least three independent technical replicates (10 identical time

	points each) using the same biochemical samples (protein, RNA) were performed. All attempts at replication were successful.<br>Cell based assays (proteomics, siRNA-mediated knock-down, RNA-seq after knock-down) were done in at least three independent technical replicates.<br>Validations of RNA-seq data for the siRNA knock-downs were done in three independent biological replicates with three technical replicates each.<br>Rescue experiments were done in three independent biological replicates with three technical replicates each.<br>UV-crosslinking and FLASH experiments were done in two independent biological replicates.
Randomization	This study reports results from rationally designed in vitro biochemical/biophysical experiments, for which randomization is not applicable, as there is no danger of confounding independent variables in the experimental design.
Blinding	This study reports results from rationally designed in vitro biochemical/biophysical experiments, for which blinding is not applicable, as the experiments did not involve human subjects, and as the results from the experiments can be objectively evaluated/quantified.

Reporting for specific materials, systems and methods

We require information from authors about some types of materials, experimental systems and methods used in many studies. Here, indicate whether each material, system or method listed is relevant to your study. If you are not sure if a list item applies to your research, read the appropriate section before selecting a response.

Materials & experimental systems

n/a	Involved in the study	
☐	☒	Antibodies
☐	☒	Eukaryotic cell lines
☒	☐	Palaeontology and archaeology
☒	☐	Animals and other organisms
☒	☐	Human research participants
☒	☐	Clinical data
☒	☐	Dual use research of concern

Methods

n/a	Involved in the study	
☒	☐	ChIP-seq
☒	☐	Flow cytometry
☒	☐	MRI-based neuroimaging

Antibodies

Antibodies used	Monoclonal FLAG-M2 antibody produced in mouse, clone M2 (Sigma-Aldrich, F1804), diluted 1:2000<br>HRP-coupled anti-His antibody produced in mouse, clone GG11-8F3.5.1 (Miltenyi Biotec, 130-092-783), diluted 1:5000<br>Polyclonal rabbit anti-BRR2 serum (kind gift of Prof. Dr. Reinhard Lührmann, Max Planck Institute for Biophysical Chemistry, Göttingen, Germany), diluted 1:500
Validation	Mouse monoclonal FLAG-M2 antibody: https://www.sigmaaldrich.com/US/en/product/sigma/f1804<br>Mouse HRP-coupled anti-His antibody: https://www.miltenyibiotec.com/US-en/products/his-antibody-gg11-8f3-5-1.html<br>Rabbit polyclonal anti-BRR2 antibody: Lauber, J., Fabrizio, P., Teigelkamp, S., Lane, W.S., Hartmann, E. & Luehrmann, R. The HeLa 200 kDa U5 snRNP-specific protein and its homologue in Saccharomyces cerevisiae are members of the DEXH-box protein family of putative RNA helicases. EMBO J 15, 4001-4015 (1996).

Eukaryotic cell lines

Policy information about cell lines

Cell line source(s)	Flp-In™ T-REx™ 293 wildtype cell line (Thermo Fisher Scientific, R78007) has been obtained from the Max Planck Institute for Molecular Genetics, Berlin, Germany.
Authentication	HEK293T is a lab stock that has been extensively used and is routinely checked for morphology. The Flp-In 293 T-REx cells have not been further authenticated after purchasing.
Mycoplasma contamination	All cell lines in our lab are routinely (monthly) tested for mycoplasm contamination by PCR. All cell lines used here are negative. PCR results are available upon request.
Commonly misidentified lines<br>(See ICLAC register)	HEK
